# Patient quality of life determinants in decision-making regarding amputation

**DOI:** 10.1371/journal.pone.0352154

**Published:** 2026-06-25

**Authors:** Carly Lightstone, Naomi Eisenberg, Sherry Harburn, Dorina Baston, Rima Styra, Graham Roche-Nagle

**Affiliations:** 1 Trilight Health, Vancouver, British Columbia, Canada; 2 Division of Vascular Surgery, University Health Network, Toronto, Ontario, Canada; 3 Division of Allied Health, University Health Network, Toronto, Ontario, Canada; 4 Department of Nursing, University Health Network, Toronto, Ontario, Canada; 5 Department of Psychiatry, University Health Network, Toronto, Ontario, Canada; Southern Medical University Nanfang Hospital, CHINA

## Abstract

**Background:**

Psychological adjustment to amputation begins well before surgery and is pivotal in determining both functional and emotional outcomes. For patients with Peripheral Arterial Disease (PAD), understanding their emotional and psychological challenges during the perioperative period is essential for healthcare teams to provide effective support. Addressing these challenges can enhance surgical outcomes and facilitate long-term rehabilitation. As patients approach amputation, their psychological adjustment significantly influences postoperative recovery and quality of life. Surgeons and healthcare teams should integrate psychological considerations into care planning to optimize outcomes and support holistic healing. This study explores the psychological challenges faced by patients with PAD undergoing amputation, providing insights relevant to surgeons in enhancing preoperative care.

**Methods:**

We conducted a qualitative study with patients who underwent primary elective amputations as part of a vascular surgery inpatient service. Data was collected through semi-structured interviews, which were conducted between five and ten days post-amputation. The interviews were analyzed using Braun and Clarke’s (2006) guidelines for thematic analysis, which allowed for the identification of key patterns and themes related to the psychological adjustment process.

**Findings:**

Three key themes emerged: *Breaking Point*, diminished quality of life threshold when patients reached a threshold of suffering from PAD; *Meaning Attributed to Amputation*, highlighting varied perceptions of the procedure’s impact on quality of life; and *Trust in the Healthcare Team*, emphasizing the role of effective communication and trust in the patient-provider relationship.

**Conclusion:**

These findings underscore the importance of addressing emotional and psychological needs of patients in the preoperative period to improve surgical outcomes. By fostering trust, and providing tailored preoperative support, surgeons along with the healthcare team can help better prepare patients to enhance post-operative recovery and reduce anxiety, and negative cognitions. Future research should explore practical effective ways to integrate psychological care into surgical practice, with a focus on improving both emotional and functional outcomes.

## Introduction

Peripheral Arterial Disease (PAD) is a leading cause of limb loss, accounting for approximately 82% of all amputations in the United States, with major amputations (both above-knee and below-knee) making up over half of the procedures performed annually. PAD is a progressive condition that severely impairs functional mobility, often resulting in pain, fatigue, and significant limitations in daily activities [1]. As PAD advances, individuals frequently experience a decline in their independence, leading to diminished capacity for engaging in daily activities and meaningful life pursuits. This can result in reduced quality of life and increased social isolation [2].

**Fig 1:**
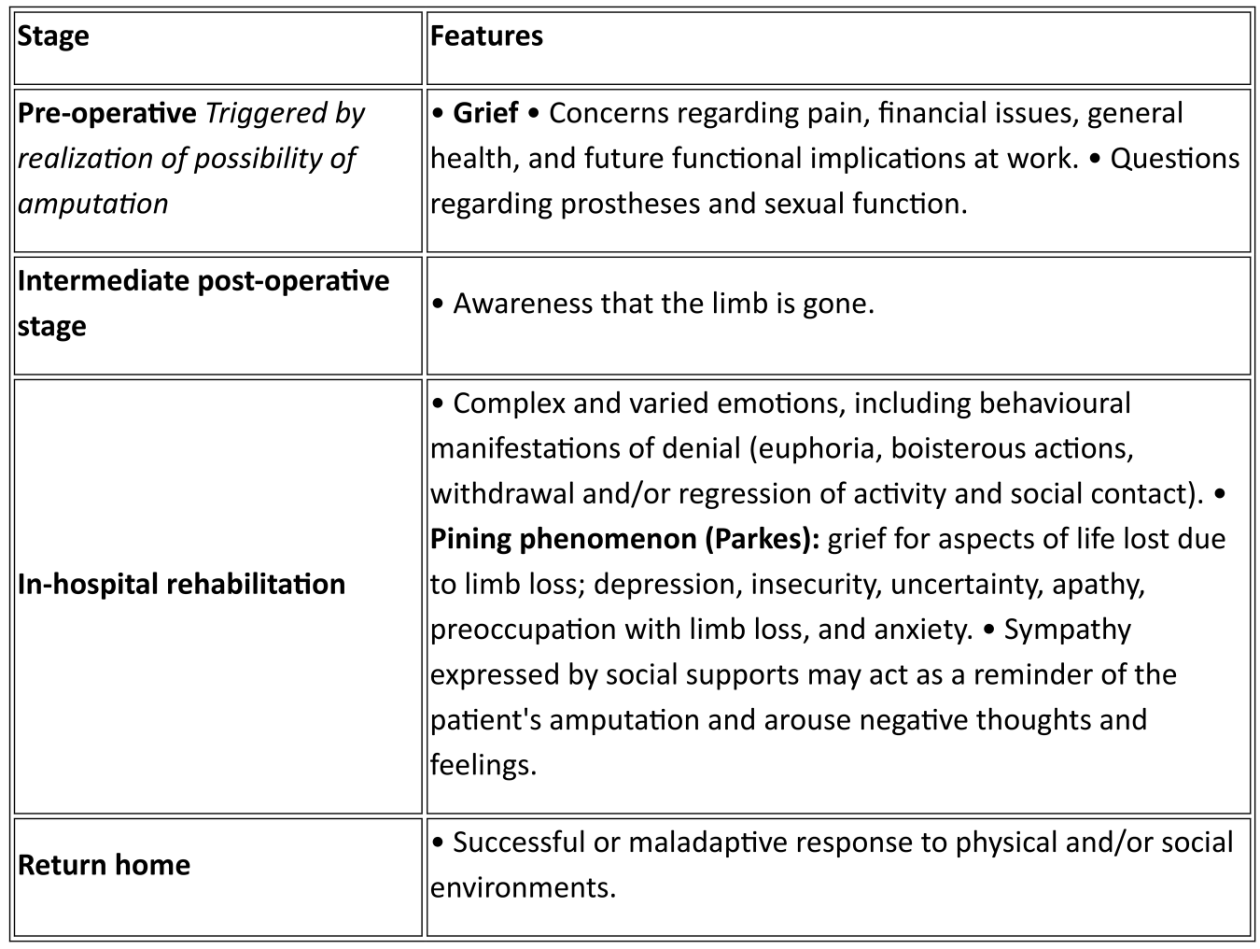
Stages of psychological adjustment to limb loss.

Amputation, while offering relief from the debilitating symptoms of PAD, introduces significant psychosocial and functional challenges. Following amputation, patients face the complex process of adjusting physically and mentally to the loss of a limb (Fig 1). The psychological burden of amputation can be substantial, affecting mental well-being, coping mechanisms, and overall adaptation to changes in functionality and independence [3].

Rehabilitation plays a pivotal role in the recovery process for patients who have undergone amputation due to PAD. A comprehensive, multidisciplinary rehabilitation team—comprising occupational and physical therapists, prosthetists, psychologists, and other specialists—addresses both the functional and psychological needs of these patients, significantly improving long-term outcomes and enhancing the reintegration process into community life [4,5]. Rehabilitation strategies have been shown to maximize mobility, reduce the risk of further complications, and improve overall quality of life [6]. However, for rehabilitation to be effective, timely and targeted interventions must begin before surgery, with emphasis placed on pre-operative psychoeducation, emotional support, and realistic expectation setting.

Psychological adjustment to amputation is a key determinant of functional outcomes post-surgery. Research has outlined four general stages of adjustment to limb loss: denial, anger, bargaining, and acceptance [7]. These stages represent common emotional responses that individuals experience as they process the trauma of limb loss. However, if these emotional reactions become extreme or prolonged, they may lead to maladaptive coping mechanisms, potentially worsening functional disability beyond what is expected from the physical loss of a limb [4].

While psychiatric involvement may be necessary in rare instances, when emotional adjustment is maladaptive the role of the healthcare team—including surgeons, nurses, and rehabilitation specialists—is crucial in providing pre-operative support. Bradway et al. emphasize that health care providers can prevent disability and reduce the need for formal psychiatric intervention through early interventions that promote psychological resilience [5]. The importance of the healthcare team’s role in providing pre-surgical support has been well documented, yet there remains a gap in literature specifically addressing pre-surgical interventions for PAD patients, particularly regarding how the healthcare team can optimize psychological preparation before amputation.

This gap in the literature highlights the need for further research to define the specific role of the healthcare team in supporting PAD patients prior to surgery, focusing on how pre-surgical interventions can influence post-operative adjustment. By gaining a better understanding of the patient’s perspective on amputation, we can inform the rehabilitation team’s pre-operative role to optimize post-operative adjustment and improve patient outcomes.

## Methods

### Study design

A qualitative research design was employed. The study was approved by the institutional Research Ethics Board.

### Recruitment and participants

A convenience sample was recruited from the acute-care vascular surgery inpatient service between January 2013- January 2015. Patients were approached sequentially and screened for eligibility. Inclusion criteria were English-speaking adults with a diagnosis of peripheral artery disease (PAD) who were scheduled for elective primary above- or below-knee amputation at our facility. Exclusion criteria included a history of major amputation, planned bilateral amputation, cognitive impairment, or emergent surgery. Participants who resided outside the Greater Toronto Area were also excluded. A total of 11 subjects consented to participate. Of the initial 13 subjects, two were not interviewed post-amputation due to complications—one participant died, and another experienced severe delirium.

### Procedure and data collection

**Fig 2:**
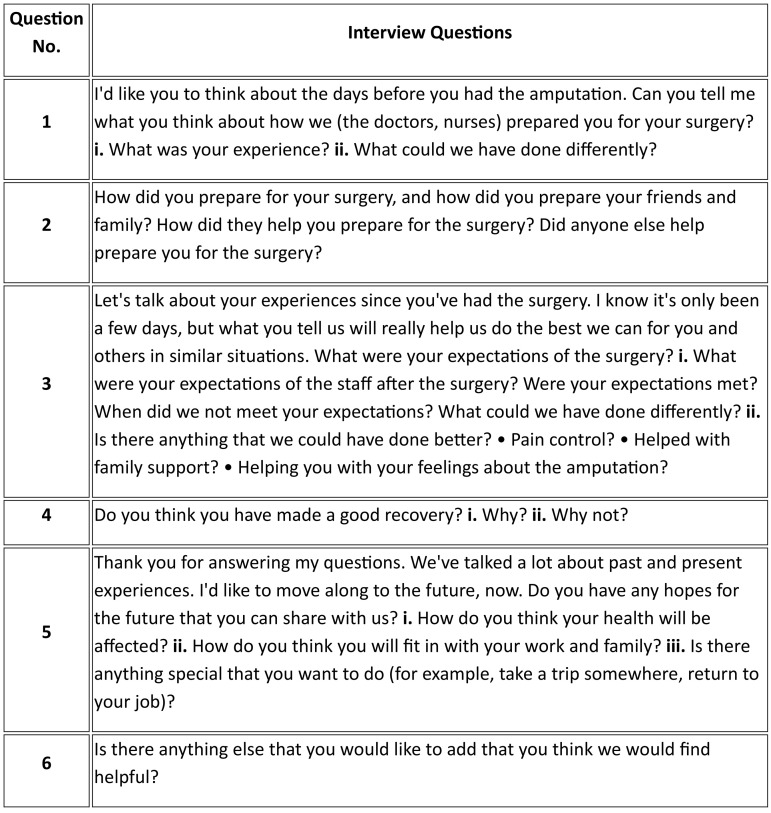
Interview questions.

Written informed consent was obtained from participants prior to surgery. Ongoing consent was re-confirmed three days post-surgery to ensure participants’ continued interest in the study. Data were collected through semi-structured interviews conducted on the vascular surgery inpatient unit, typically five to ten days after amputation. Each interview lasted approximately one hour. All interviews were audio-recorded by the interviewer and transcribed verbatim by a professional transcriptionist. The interviewer used an interview guide with six open-ended questions to structure the conversation, while also allowing for unplanned follow-up questions to explore emergent themes (Fig 2). All interviews were conducted by a co-investigator who was not involved in the participants’ clinical care.

### Data analysis

Interviews were analysed in accordance with Braun and Clarke’s guidelines for thematic analysis [8]. Thematic analysis is a systematic method in qualitative research used to identify, analyse and report themes within a data set. An inductive approach to thematic analysis was employed, whereby the data itself, rather than a pre-existing theoretical framework drove the emerging themes [9]. In keeping with the guidelines for thematic analysis, each interview transcript was independently reviewed and coded by investigators, and the interesting features of the data were organized into initial codes [8]. Three to five authors then worked together to enhance rigour and a multidisciplinary perspective. Codes were then further reviewed and grouped into potential themes. Potential themes and related subthemes were reviewed and checked in relation to codes, and a thematic map was created. Finally, the themes and subthemes representing the key factors related to patients’ pre-operative perceptions relating to psychological adjustment were defined and named by three investigators. Peer debriefing was undertaken with an external researcher who has extensive experience conducting qualitative research.

## Results

Table 1 summarizes the demographic characteristics of our sample, which are typical for a population with peripheral artery disease (PAD), including a history of diabetes, cardiac disease, hypertension, and smoking.

**Table 1:** Patient characteristics. N = 13 (%).

Age (Range = 52–71)	Avg. = 62.7
Male	7 (53.8)
Smoking History	9 (69.2)
Diabetes	9 (69.2)
Hypertension	10 (76.9)
Renal Failure	2 (15.4)
Cardiac Disease	5 (38.5)

Three key themes emerged from the data: 1) Breaking Point, 2) Meaning Attributed to Amputation, and 3) Trust in the Healthcare Team. Each of these themes, along with their corresponding subthemes, is discussed in greater detail below.

### 1. Breaking point- diminished quality of life

When discussing their experiences with PAD prior to amputation, participants highlighted the progressively severe and debilitating impact of the disease on their daily lives, function and overall quality of life, where they could no longer cope and the benefits of undergoing an amputation outweighed the prospect of continuing to live with the disease. Several factors were identified in the following subthemes:

#### a. Disease management.

Participants described a rapid increase in the number of hospitalizations for disease management or limb salvage. They described their quality of life as becoming negatively impacted that they felt ready for amputation. One participant stated, “I was in the hospital, in Emerg, 4 to 5 times a month, every month, for 8 months”. [Participant 2] Another participant expressed frustration with repeat hospital visits: “I’m ready for it, because I was just totally fed up… I was getting tired of coming into the hospital all the time. “[Participant 6].

Others spoke of difficulty navigating the healthcare system in order to receive treatment for their health concerns, describing the burden of misdiagnoses and the minimization of symptoms by clinicians:

… I went to another hospital 2 years ago and they told me there was nothing wrong… so I ended up coming here, ended up here for three weeks… I went to (a different) Hospital... and I said I have pain, I’m a diabetic I have pain. They told me to go home, so I went home and then I walked into the drugstore, the drugstore owner he called the ambulance, and he brought me here and I stayed in this hospital for three weeks. [Participant 1]

#### b. Sensory symptoms.

Participants explained the changes in sensation, appearance and odor as specific markers of the disease progression.

…circulation …in the foot wasn’t there because it felt like it was thirty below…when you’re standing on a hot heating pad and you don’t even feel it, I’m lucky I didn’t burn my foot…It felt like it was frozen…I was that swollen that that’s what caused me to make up my mind was the water blisters. It was bad. [Participant 13]

*…*my foot was all black…water and pus it was terrible, you had to plug your nose. [Participant 1]

Participants were also aware that when their health deteriorated to a critical point, there was no hope for recovery of their lower-limb.

This one little toe, the second and the third toe had already died, no blood coming in, it was just blocked, dead... blood wasn’t getting down there; it was like a cage, nothing in there, just the rib, just the cage. I realized that... I can’t grow back meat on something that is no longer there. [Participant 7]

#### c. Pain threshold.

The majority of participants reported reaching a threshold at which the severity and persistence of pain rendered it unmanageable.

The pain was so bad in my foot. So, I went upstairs and I spoke to the lady at the front desk, the receptionist or whatever you call it. ‘Ma’am I’m sorry’, and I was crying, I had tears in my eyes. I said ‘I can’t take the pain anymore, I need to see the doctor urgently, I can’t take this pain’. [Participant 1]

Participants reflected the degree of pain by recounting their pharmaceutical regimen:

“Like there’s only so many painkillers you can take and it’s worse when you have a high resistance to drugs, like me. You end up – give me more morphine, and I’m starting to get immune to it and it’s getting to the point where it’s not working.” [Participant 13]

For some the prospect of an amputation represented freedom from pain and its disabling effects.

…well I expected to have pain, I mean you have surgery, you’re going to have pain. I knew I was going to have pain but I’ve been having pain for 8 months and I thought, I felt it was never going to be any kind of pain like that, ever again. [Participant 2]

“I was actually happy I lost the pain – they take my pain, not take my leg, take my pain.” [Participant 11]

#### d. Care partner burden.

Several participants’ indicated that their breaking points were significantly influenced by the escalating increased care partner demands placed on their care partners as a result of the diseases due to the disabling effects of the disease. One participant described the impact on the life of her care partner.

My daughter was doing all my banking, all my shopping, and it was getting to the point where it wasn’t fair to her. She didn’t have a life anymore... My daughter, she said ‘mom I am fed up myself’, she said ‘sorry mom, but I’m getting tired of helping out’. She said ‘I don’t mean to be hateful’. She said ‘I’m getting so tired of doing all your running around’. [Participant 6]

For this participant, a motivating factor in the decision to undergo an amputation was her desire to lessen the burden that caregiving placed on her family member.

### Summary

Participants described PAD as progressively debilitating, severely diminishing their function and quality of life to the point where amputation was viewed as preferable to continued disease management. Frequent hospitalizations, difficulties accessing appropriate care, and perceived misdiagnoses contributed to frustration and readiness for amputation. Worsening sensory changes, visible tissue deterioration, and the realization that limb recovery was no longer possible reinforced this decision. Unrelenting pain that became resistant to medication was a critical tipping point, with many viewing amputation as relief from suffering. Additionally, concern about the growing burden placed on family caregivers influenced participants’ decisions, as amputation was seen as a way to reduce strain on loved ones.

### 2. Meaning attributed to amputation

A second theme that emerged was the meaning that participants attributed to the prospect of undergoing and subsequently living with an amputation. Participants voiced concerns around the restructuring of social roles, self-concept and values after undergoing amputation. Three subthemes related to the meaning that participants attributed to amputation are described below:

#### a. (Un) familiar future.

Several participants felt that they would have been better prepared for amputation had they received more information regarding post-operative expectations:

Well I don’t, just, what’s it like after you have the amputation, how are you expected to feel, just, or, you know, like... I wasn’t told anything, just that my leg was going to be amputated, nothing else other than that. You know, that’s a scary thing. Somebody should have... could have told me what it’s going to be like after. [Participant 2]

I was so scared. Unbelievably scared... I didn’t know what’s going on, how it will be so and so. [Participant 3]

For these participants, an unfamiliar future caused fear prior to amputation. In contrast, another participant described a sense of clarity and reduced fear having witnessed others’ experience of amputation.

Well I’ve seen friends have amputations like mine and I see them moving around and getting around, I mean not as easy as with two feet. So I said to myself it’s not the end of the world, getting an amputation, I’m not the first one or the last one that’s going to get it. [Participant 7]

#### b. (In) dependence.

The prospect of undergoing an amputation represented a change in independence for participants despite their previous level of functioning. Many participants conceptualized amputation as the loss of independence and progression towards helplessness.

[I thought about] what I wasn’t going to be able to do when it happened, I just thought about all the things that I wouldn’t be able to do. Well, I wasn’t doing much anyways but I could at least try to do something when I had my leg; I could walk on my heel and stuff, I could get from my bedroom to my bathroom, now I can’t do anything. I’m helpless, and I don’t like that. I was very independent before and I’m still independent. [Participant 2]

This patient identified the dissonance between the impact of amputation on independence and the internal struggle of re-establishing ones’ identity. Others viewed amputation as an opportunity to achieve greater independence due to the relief of disease symptoms.

No, I kind of figured we’d go into prosthetics and so it’s easier to walk around with little pain from prosthetics than walk around with the pain that I had. Of course, I wouldn’t have been able to walk around with the pain that I had because I wouldn’t last too long. [Participant 13]

#### c. Perception of self within family.

For some participants it was important that their family and/or friends demonstrate comfort and approval with their decision to undergo amputation. Support from family was related to acceptance of living as an amputee within the family unit. Participants described conversations with family prior to the amputation that provided reassurance and strengthened their confidence in their decision.

They said ‘okay mom, if you feel you need to have it off so we can have you a lot longer, then go ahead, go and take it off’. [Participant 6]

Well they talked to me and stuff and said ‘you know nobody’s going to love you any less with one limb’. [Participant 2]

### Summary

Participants described amputation as a major life transition that reshaped their sense of self, future, and social roles. Fear and anxiety were common when the future felt unfamiliar, particularly when participants lacked information about post-amputation life, while exposure to others’ successful experiences reduced fear and increased acceptance. Amputation was often associated with anticipated loss of independence, though some participants viewed it as a pathway to greater independence through pain relief and prosthetic use. Acceptance was also strongly influenced by family relationships, with reassurance and approval from loved ones helping participants feel valued and supported within the family after amputation.

### 3. Regard for the healthcare team

Many participants discussed their experiences with the healthcare system, highlighting both positive and negative interactions with providers. Years of interaction with clinicians had an impact on their acceptance of their amputation, as represented by the following subthemes:

#### a. Timeliness.

We found that participants who had less time to prepare for their surgery had more difficulty accepting their amputation.

When I went to the doctor’s office for an appointment, he came in and took one look at my leg and he said ‘In the hospital’ to the other doctor ‘look’ to the other doctor, and he said ‘we are going to have to amputate’ and I just looked at him and he said ‘will tomorrow be good enough?’ [Participant 2]

This participant continued to describe her reaction to this news, stating “I was down, depressed, just crying all the time, just trying to get it into my head and just I was a basket-case”. [Participant 2] The urgency of being informed about her need for amputation impacted her ability to process and accept the reality of the situation. Conversely, participants were frustrated with the healthcare system once they had reached the point of accepting their imminent amputation but were left suffering while waiting days for the procedure.

Participants also discussed the detrimental effects of timeliness of healthcare services as this factor had significant bearings on health outcomes.

… but putting blood down there where it needed it was a little bit too late. Maybe if it was done a month earlier, rather than cut off the whole thing they would cut off only two or three of the toes. [Participant 7]

#### b. Transparency.

Participants seemed to hold high regard for healthcare providers who were open and honest about their health condition and health outcomes. This was important for participants in accepting and understanding information.

I mean being open, was very good. Being told what was going to be done to me, Dr.___ did a very good job of that. He did a very good job. I didn’t even have to ask when I came back here, I didn’t even have to ask him anything and he did come that morning. [Participant 4]

A factor that appeared to have an impact on participants was being provided with detailed information regarding the amputation, specifically information such as above or below knee).

So, they prepared me and took me home and ‘we’re going to try to see’, they tell me plain, ‘more likely we have to amputate below the knee, if there is any possibility that lower down then we’ll try to help you, but prepare for the knee’. [Participant 7]

#### c. Compassion.

Participants had greater trust in healthcare providers who built rapport with them and care for them on a personal level rather than simply providing them with physical care.

You didn’t push me. The doctors told me even in the downstairs that room before you go to surgery... they were so nice with me. They told me because they saw I was so sad, but they didn’t try to do anything so they asked me if I wanted to go through with this, and I said I have to because I got gangrene. [Participant 3]

In contrast, participants voiced disappointment when healthcare providers lacked compassion.

“Just the way he put it, ‘will tomorrow be good enough?’... my ex-husband said like ‘where’s the compassion?’ just ‘we got to amputate, is tomorrow good enough?’ just like that.” [Participant 2]

Participants appreciated healthcare providers who seemed to go “above and beyond”. One participant described the concern demonstrated by his physician. “When I went home, he would phone me every once in a while, and asked me how my diabetes was going.” [Participant 1]

Many participants said that they were moved by gestures of compassion received from healthcare providers, as they represented a source of additional support throughout the pre-amputation process.

#### d. Psychological support.

The majority of participants did not receive professional psychological support. Ongoing informal support from the interdisciplinary team on the vascular surgery unit was provided with mental health consultations required as needed. Those who felt they had support from their healthcare providers reflected more positively with respect to their views of their amputation. “The nurses and the doctors here came and look at me and encouraged me and you feel that you’re not alone.” [Participant 7] One participant talked about a negative experience with one doctor, leading her to feel unsupported and mistrustful of him. In contrast, she spoke positively about the support she received from her subsequent physicians, demonstrating that increased psychological support can lead to increased satisfaction with healthcare and the overall pre-amputation experience.

Well I didn’t get any support from one particular doctor, I wouldn’t want to have that doctor again if you paid me... but people have asked me over the course of two years, why didn’t he give you more support? And I said I don’t know…. But any other doctor that has stepped in to help me out were fantastic to help me [Participant 6]

Patients voiced that increased psychological support from staff would have been a beneficial resource in preparation for amputation. “Somebody could have come in to talk to me... It was extremely hard. I felt like I went through it by myself.” [Participant 2].

### Summary

Participants’ interactions with the healthcare system strongly influenced their acceptance of amputation. Limited time to prepare for surgery and delays in care made acceptance more difficult and were perceived as negatively affecting outcomes. Transparent communication helped participants understand and prepare for amputation, particularly when providers clearly explained the procedure and possible outcomes. Compassionate, rapport-based care increased trust and emotional comfort, while abrupt or insensitive communication led to distress and dissatisfaction. Although most participants did not receive formal psychological support, those who felt emotionally supported by healthcare providers reported more positive experiences, and many expressed that greater psychological support would have improved their preparation for amputation.

## Discussion

Our study offers significant insights into the emotional and psychological challenges that peripheral artery disease (PAD) patients face in the peri-operative period, particularly those undergoing amputation. The findings lend insight to the quality and conversational approach between healthcare providers (who could be a surgeon or the nurse) and patients. It also underscores the necessity of a comprehensive, multidisciplinary approach to pre-surgical care that involves surgeons, psychologists, nurses, and other healthcare providers. While surgeons remain central to the surgical process, their role as the central most responsible physician, includes ensuring appropriate resources to manage patient expectations through pre-surgical evaluation and facilitating early psychological support as required. The findings also allow us to shine a light on team members’ behaviours that may affect those relationships.

The central theme that emerged from our study is the concept of a “*Breaking Point*” emerged as a pivotal theme in understanding the decision-making process of PAD patients. This refers to the emotional and physical quality of life threshold at which patients believe amputation is their only viable option, driven by worsening symptoms such as pain, somatic symptoms, and mobility limitations. Surgeons can play a critical role by recognizing when a patient is approaching this threshold, initiating discussions, and providing clear, honest, and timely communication regarding the surgical option. Early recognition of this emotional turning point can enable surgeons to ensure timely psychoeducation and facilitate the patient’s emotional readiness for surgery. Studies suggest that early involvement of healthcare professionals in these discussions improves patient satisfaction and surgical outcomes [10,11].

The decision-making process is significantly influenced by factors such as the burden of disease management, the need for care partner support, and the patient’s quality of life. While previous studies on amputation populations emphasize the role of social participation [12], our study found that social participation did not emerge as a significant factor, likely due to the chronic nature of PAD, which already limits patients’ social engagement. This insight is significant for surgeons who should recognize that social isolation may not be a priority in the pre-operative phase, rather, the focus should be on quality-of-life improvements post-amputation [13].

The “meaning attributed to amputation” is a complex issue, with patients varying in their perceptions from viewing amputation as a relief from ongoing pain to fearing a loss of independence and self-identity. Surgeons must be aware of these differing perceptions and ensure both practical and emotional support. Prior research underscores that pre-surgical psychoeducation can significantly reduce pre-operative anxiety and help patients develop realistic expectations [14]. Our findings suggest that psychoeducation can help patients prepare for the post-operative recovery period by addressing fears, explaining the rehabilitation process, and managing expectations about functional outcomes [11]. For PAD patients, providing information on expected functional recovery and prosthetic use (where applicable) can mitigate the fear of the unknown and contribute to a more positive outlook.

Additionally, surgeons should also facilitate connections with peer support groups or individuals who have undergone similar surgeries. This can improve the patient’s mental preparedness by providing concrete examples of successful rehabilitation and adjustment. Studies have shown that peer support can significantly reduce the emotional burden of surgery and foster a sense of hope [15].

Our study identified “*regard for healthcare*” as a crucial factor in how patients perceive their surgery and the care they receive. Trust in the healthcare team, as well as transparency in communication, significantly influences patients’ acceptance of amputation and their psychological preparedness. Surgeons must be proactive in offering clear, honest, and compassionate communication, which has been shown to improve psychological resilience and the patient’s trust in their healthcare providers [10]. Furthermore, research indicates that non-sugar-coated communication, which presents a realistic view of the surgery and its potential complications, fosters a stronger patient-surgeon relationship and improves patient satisfaction with care [16].

While our study highlighted the importance of pre-operative psychoeducation, we also observed gaps in formal psychological support. This aligns with the findings of previous studies, which suggest that many patients experience a lack of psychological support during the pre-operative period [11]. Surgeons can help bridge this gap by ensuring that psychoeducation is incorporated into pre-surgical consultations. Surgeons should work closely with interdisciplinary healthcare teams, including psychologists, psychiatrists and social workers, to provide comprehensive care that addresses the psychological, and physical aspects of the surgery [10,14].

Family members, who often act as the care partners, need to be involved in pre-surgical discussions as an essential component of emotional support for the patient. Surgeons can encourage patients to involve family members early in the decision-making process, as this has been shown to improve both psychological outcomes and post-operative recovery [17]. Involving family members in psychoeducation sessions can reduce feelings of social rejection and isolation and help the family members prepare for the changes in social roles and responsibilities post-surgery [12].

One of the benefits of qualitative research is that it allows the team to generate opportunities for targeted interventions, professional reflection and development as well as ongoing research.

There are several limitations to this study. It was conducted at a single-center with a small sample size, limiting its generalizability to broader populations. We did not control for individuals with pre-existing mental health diagnoses. Furthermore, the study did not explore cultural influences or individual differences in coping strategies. Future studies should expand the sample size and consider including participants from different cultural backgrounds and exploring personality traits or coping styles, as these may influence the psychological outcomes of PAD patients undergoing amputation.

These findings have significant implications for healthcare providers, particularly vascular surgeons and their multidisciplinary teams. A pre-operative understanding of a patient’s emotional state can play a pivotal role in shaping the patient’s expectations and outcomes. By addressing emotional needs early in the treatment process, healthcare providers can help patients make more informed decisions, facilitate emotional preparation and enhance their psychological resilience. Fostering a trusting relationship with patients before the surgery can also lead to better post-operative outcomes, as patients are more likely to follow through with rehabilitation, experience lower levels of distress, and exhibit greater satisfaction with their care.

Furthermore, the themes identified in this study underscore the importance of a patient-centered approach to care, in which healthcare providers consider not only the physical aspects of the amputation but also the emotional, and social factors that affect the patient’s experience. Ensuring that patients feel heard, supported, and informed before and after the procedure can have lasting positive effects on their recovery and quality of life.

Future research should explore strategies to integrate these findings into practice, particularly by developing interventions or psychoeducation programs tailored to the unique psychological needs of PAD patients undergoing amputation. Pre-surgical psychological screening and counseling may also be valuable components of the care process, helping to address potential psychological challenges before they hinder recovery.

## Conclusion

This study highlights the critical importance of addressing both the physical and psychological needs of PAD patients in the pre-operative period. Surgeons, along with the healthcare team, play a critical role in initiating early communication about the potential need for amputation and offering pre-surgical psychoeducation to prepare patients emotionally. Incorporating psychological support and encouraging the involvement of family or care partner can significantly improve the patient’s ability to cope with the surgery and recovery, ultimately leading to better surgical outcomes and enhanced quality of life.

## Supporting information

S1 File Inclusivity-in-global-research-questionnaire. (DOCX)
