# Supplementary material for: Patient quality of life determinants in decision-making regarding amputation
Source: PLoS One. 2026 Jun 25;21(6):e0352154. doi: 10.1371/journal.pone.0352154 (PMC13298963; doi:10.1371/journal.pone.0352154)
Supplement: Attachment — Submitted filename: PLOS Reviewers response Jan 5.docx [file pone.0352154.s004.docx]

**Comments to the Author**

4. Have the authors made all data underlying the findings in their manuscript fully available?

The [PLOS Data policy](https://urldefense.com/v3/__http:/track.editorialmanager.com/CL0/http:*2F*2Fwww.plosone.org*2Fstatic*2Fpolicies.action*23sharing/1/010f019b45266d63-ce19a144-b821-4d6a-be31-0598ee22c9be-000000/gOdxacCcpdwmL_DhfZxbRsKPAwSPzgMhcrb8X57CXio=242__;JSUlJSU!!CjcC7IQ!MEfJ3Pf9puzR_R0tBkaw7T1q2twgO_mScfkMEBNcR8XkuhCjEUpbvXAtpihqFljCmQdpoBG-tNtsDOb3CLJ1zFo$) requires authors to make all data underlying the findings described in their manuscript fully available without restriction, with rare exception (please refer to the Data Availability Statement in the manuscript PDF file). The data should be provided as part of the manuscript or its supporting information, or deposited to a public repository. For example, in addition to summary statistics, the data points behind means, medians and variance measures should be available. If there are restrictions on publicly sharing data—e.g. participant privacy or use of data from a third party—those must be specified.

Reviewer #1: No

Reviewer #2: Yes

**The base data are the interview transcripts with some potential patient identifiers hence not shared for participant privacy. There are no summary statistics, data points behind means, medians and variance measures.**

6. Review Comments to the Author

Please use the space provided to explain your answers to the questions above. You may also include additional comments for the author, including concerns about dual publication, research ethics, or publication ethics. (Please upload your review as an attachment if it exceeds 20,000 characters)

Reviewer #1: Thank you for addressing my previous comment and implementing the requested adjustment. I appreciate the swiftness with which you have incorporated this feedback.

- Minor typographical correction needed
While reviewing the revised text, I identified a few minor typographical errors that should be corrected for publication. For instance, the phrase: "Individual in-depth interview" is likely intended to be formatted as: "individual"

I kindly request that you perform a thorough check of the text for any remaining spelling or spacing discrepancies to ensure the final manuscript is polished and accurate.

**Corrected**

Reviewer #2: The paper is very well-written, and all comments from the previous reviewer were addressed. I feel the interpretations are valid. The only minor issue is that many of the sections in the results rely on quotes to end them. I would like to see more of an integration/summarizing of what the described issues/ideas - similar to what is presented in the compassion sub-theme. I feel it would really elevate the quality of the findings if this was adopted more throughout the results section. Otherwise, I feel everything else has been done very well.

**Summary added to results sections**

Minor point - you spell out PAD twice in the abstract.

**Corrected**
