# Supplementary material for: Patient quality of life determinants in decision-making regarding amputation
Source: PLoS One. 2026 Jun 25;21(6):e0352154. doi: 10.1371/journal.pone.0352154 (PMC13298963; doi:10.1371/journal.pone.0352154)
Supplement: Attachment — Submitted filename: Reviewer responses.docx [file pone.0352154.s003.docx]

Reviewer #1: General comments:
I would like to congratulate the authors on a well-designed and valuable study. The manuscript adds a crucial perspective to the existing literature on psychological adjustment following amputation. Its primary strength lies in the qualitative methodology, and the findings/themes identified, providing a rich and in-depth understanding on these matters.

Specific comments:
1. Title and abstract
The terms "Quality of life determinants" and "Psychological adjustment" are used interchangeably throughout the title and abstract. For enhanced clarity and alignment with the study's findings, I recommend focusing on the core theme of "Psychological adjustment." A consistent use of this terminology will improve the overall coherence and precision of the manuscript.

**Thank you. I will change to Psychological adjustment.**

2. Methodology
• More specific details about the interview guide/interview question are recommended in the ‘Methods’ section. To enhance transparency and reproducibility, I recommend either providing a list of the six open-ended questions in the main text/table or including a supplementary document with the full interview guide.

**Thank you. I will add the open end questions – Figure 2**

• Please explicitly clarify whether the data was collected via focus groups or individual in-depth interviews. This distinction is critical as each method influences the type of data generated, and this information is necessary for readers to properly evaluate the research design.

**These were Individual in-depth interviews. These were transcribed by a person not involved with the patients.  Study members then met and reviewed the interviews, identified themes and categorized them.**

• The manuscript notes that interviews were conducted 5-10 days post-amputation. Please provide a brief rationale for selecting this specific timeframe. Justifying this decision will strengthen the methodological rigor of the study.

**We aimed to interview patients at a point late enough in the acute post-operative phase for them to be cognitively able to participate, when they had begun mobilizing and had adequate pain control, but before their transition to rehabilitation**
